# Therapeutic potential of *Buddleja Polystachya* Fresen (stem and leaves) extracts: antimicrobial and cytotoxic properties for ocular disease management

**DOI:** 10.1007/s12672-024-01138-2

**Published:** 2024-07-15

**Authors:** Ali Hendi Alghamdi, Asaad Khalid, Aimun A. E. Ahmed, Haidar Abdalgadir, Mahadi Bashir, Ashraf N. Abdalla, Sami S. Ashgar, Hamdi M. Alsaid, Magbool E. Oraiby

**Affiliations:** 1 Surgery Department, Faculty of Medicine, Al Baha University, P. O. Box: 1998, Al Baha, 65515 Saudi Arabia; 2 Health Research Center, Jazan University, P. O. Box: 114, Jazan, 82511 Saudi Arabia; 3 Pharmacology Department, Faculty of Medicine, Al Baha University, P. O. Box: 1998, Al Baha, 65515 Saudi Arabia; 4 Pharmacology Department, Faculty of Pharmacy, Omdurman Islamic University, P. O. Box 2587, Khartoum, 11111 Sudan; 5 Biology Department, Faculty of Science, Al Baha University, Al Baha, 65515 Saudi Arabia; 6 Department of Pharmacology and Toxicology, College of Pharmacy, Umm Al-Qura University, Makkah, 21955 Saudi Arabia; 7 Department of medical microbiology, Faculty of medicine, Umm Alqura University, Makka, 21955 Saudi Arabia; 8 Poison Control and Medical Forensic Chemistry Center, Jazan Health Affairs, Ministry of Health, Jazan, Saudi Arabia

**Keywords:** *Buddleja Polystachya Fresen*, Natural phytochemicals, GC-MS analysis, Ocular infections, Antimicrobial activity, Cytotoxicity, MCF7, HT29, HepG2

## Abstract

This study on *Buddleja polystachya* highlights its phytochemical composition, antimicrobial activity, and cytotoxic impacts. The study emphasizes the plant’s potential to treat ocular diseases by identifying important compounds involved in the bioactivity through GC-MS analysis. This study explores the antimicrobial and cytotoxic potential of *Buddleja polystachya* (stem and leaves) extracts, with a focus on their application in treating bacterial ocular infections and their efficacy against MCF7, HT29, and HepG2 cancer cells. Through comprehensive GC-MS analysis, a diverse array of phytochemicals was identified within *Buddleja polystachya* stem and leaves extracts, including carbohydrates, phenolic derivatives, fatty acids, and steroidal components. The extracts were then evaluated for their biological activities, revealing significant antimicrobial properties against a range of bacterial strains implicated in ocular infections. The research findings demonstrate that stem extracts derived from *Buddleja polystachya* demonstrated high to moderate cytotoxic effects on cancer cell lines MCF7, HT29, and HepG2. Notably, these effects were characterized by varying IC_50_ values, which suggest distinct levels of sensitivity. In contrast, leaf extracts exhibited reduced cytotoxicity when tested against all these cell lines, although they did so with a significantly higher cytotoxicity aganist HepG2 cells. The results of this investigation highlight the potential therapeutic utilization of *Buddleja polystachya* extracts in the management of ocular infections and cancer. These results support the need for additional research to elucidate the underlying mechanisms of action of these extracts and explore their potential as drugs.

## Introduction

Finding medicines for eye problems that work efficiently without causing adverse effects is a major challenge in the medical field as conventional chemical treatments frequently have narrow therapeutic window and side effects. On the other hand, medicinal plants provide a viable alternative to traditional treatments because of their effectiveness, less side effects, and affordability [1]. Treating eye problems with effective and side-effect-free drugs is still a challenge for the medical field that can be surpassed by the medicinal plant-based drug discovery since natural products-based drugs can overcome most of the limitations of the conventional medications. The exploration of herbal medications has led to the study of over 200 medicinal plants worldwide, aimed at managing complications associated with eye conditions. Interestingly, some of these plants have been acknowledged and endorsed by the Indian traditional medicine system for their healing properties [2, 3].

Medicinal plants, known to chemists and pharmacologists, as ‘’crude pharmaceuticals of natural or biological origin’’ are complete plants or portions of plants with therapeutic characteristics. [4]. One of these plants is the remarkable *Buddleja polystachya* Fresen, (*B. polystachya*) also called “Afar” in Arabic. This plant, indigenous to Saudi Arabia and abundant in its southern area [5], has historically been utilized in Ethiopia and other East African nations for the treatment of many diseases, including skin conditions, and eye-related conditions [6, 7]. *B. polystachya* is traditionally used by topically applying crushed fresh leaves, resulting in a considerable reduction in wound areas according to previous studies [8, 9].

Previous research on *B. polystachya* has validated its traditional uses and uncovered a wider range of biological properties. Twenty compounds were isolated from this plant have shown adulticidal efficacy against *(A) aegypti* mosquitoes, while its hydro-alcoholic extract has demonstrated antidiarrheal and antispasmodic activities [10, 11]. Despite these interesting results, there is still a considerable lack of information in the literature about the phytochemical composition *of (B) polystachya*, especially by modern automated chemical analytical techniques such as GC-MS, which could provide information about its volatile and semi-volatile components.

This study intends to fill this gap by phytochemical profiling of *B. polystachya*, using automated GC-MS analysis for the first time up to our knowledge. This work involves assessing the extract’s antimicrobial properties and cytotoxic effects on different human cancer cell lines (MCF7, HT29, and HepG2). This detailed analysis explores the stem and leaf extracts of *B. polystachya* and the use of contemporary scientific methods to explore its therapeutic characteristics that could lead to the formulation of new herbal products for ocular health.

## Materials and methods

### Phytochemical studies

#### Plant material identification, collection and extraction

*B. polystachya* plant was collected from Baljurashi province, Al Baha, Saudi Arabia in April 2021. The plant was identified by taxonomist Dr. Haidar Abdalgadir at the Department of Biology, Faculty of Sciences, University of Al Baha. Voucher herbarium specimen number (BUH-7X) were deposited at the herbarium of the Faculty of Science, University of Al Baha, Saudi Arabia. Each of the stem and leaves of the plant was extracted using a slightly modified procedure previously described by Harborne [12]. Briefly, the plant materials (250 g each of stem and leaves) was shade-dried, powdered, and macerated in 80% ethanol v/v for one week at room temperature. The extracts were filtered, evaporated to dryness, and the yield percentage was calculated using the formula: Yield % = (afforded extract weight)/ (air dried weight) x100.

#### GC-MS analysis

We conducted analyses using a Shimadzu Gas Chromatograph (Shimadzu, Japan) with a TR-5MS (30 m × 0.25 mm) capillary column and a Shimadzu QP2010 Ultra MS detector. Helium was the carrier gas. The temperature program started at 70 °C, increased by 15 °C/min to 300 °C, and held for 30 min. Fragments were detected using Shimadzu QP2010 Ultra MS detector coupled to the chromatograph instrument with electron-ionization system and ionization energy of 70 eV. Ion source temperature was maintained at 230 °C. Constituents were identified by comparing retention indices and fragmentation patterns with authentic standards and library databases.

#### Identification of the plant constituents

The plant’s constituents were identified using authentic standards (Sigma Aldrich, Darmstadt, Germany) by comparing their retention indices. The retention indices and the fragmentation pattern which were in close agreement to reference standards were used for identification. For other constituents, the fragmentation patterns were compared and identified using in-built NIST08 and Wiley 9 libraries stored in the software database. The area % values were used for quantification of the constituents.

### Antimicrobial studies

#### Microbial strains

In this study we focused on common bacteria causing ocular infections, i.e. *Streptococcus pneumoniae* (ATCC 49,619), *Staphylococcus aureus* (ATCC 25,923), *Pseudomonas aeruginosa* (ATCC 15,442), *Haemophilus influenzae* (ATCC 10,211), *Neisseria gonorrhoeae* (ATCC 43,069), in addition to one fungal clinical isolate *Candida albicans*.

#### Culture and susceptibility testing

Cultures were grown on Muller Hinton agar, Nutrient agar, Blood agar, Chocolate agar, Sabouraud Dextrose agar, Muller Hinton broth media, and incubated at 37 °C for 24 h. The agar well diffusion method was used to assess antimicrobial activity, with wells cut into agar plates and filled with the extracts. The diameters of inhibition zones were measured after 24 h.

#### Determination of MIC and MBC

The Minimum Inhibitory Concentration (MIC) and Minimum Bactericidal Concentration (MBC) were determined for the selected extracts against *S. aureus* and *P. aeruginosa* using a broth dilution method in 96-well microtiter plates.

#### Preparation of standard inoculum

*S. aureus* and *P. aeruginosa* were grown on Muller Hinton agar, and *S. Pneumoniae* grown on blood agar, *H. influenzae, N. gonorrhoeae* grown on chocolate agar, and *C. Albican*s grown on sabouraud dextrose agar all at 37 °C for 24 h. Colonies were selected by sterile loop and inoculated into Muller Hinton broth to form homogenous suspension of test bacterial strain which was standardized to 0.5 McFarland using calibrated Vitek Densichek Biomerieux Analyzer.

#### Diffusion assay on agar plates

The agar well diffusion method was was performed according to a previous method [13]. Test bacterial strains were suspended in Mueller Hinton broth and adjusted to 0.5 McFarland turbidity. They were swabbed on Mueller Hinton agar, blood agar, chocolate agar, and sabouraud dextrose agar plates using sterile cotton swabs. The plates were dried, then cut into wells of 6 mm diameter. Each well was filled with 100 µL of the tested extract, a positive control for Gram-negative bacterial strains, Amikacin (30 µg Disc, eye inection antibiotic), Vancomycin (30 µg disc) were used as standard drugs, while dimethyl sulfoxide (DMSO) was used as a negative control at non toxic dose. The technique was repeated two times and incubated for 24 h at 37ºC. The plates were examined and the diameter of the inhibition zone was measured and recorded for each extract.

#### Determination of the MIC and MBC

The Broth Microdilution Method was used to determine the Minimum Inhibitory Concentration (MIC) and Minimum Bactericidal Concentration (MBC) of *B. polystachya* extract. The assay involved adding 200 µl of each extract at a concentration of 100 mg/ml followed by 100 µl of Mueller Hinton broth. The extracts were then diluted in a two-fold order, and 10 µl of 0.5 adjusted McFarland bacterial suspension was added to all wells. The technique was repeated two times simultaneously in microtiter plates, and the plates were incubated at 37 ºC overnight. The MIC was recorded as the minimum inhibitory concentration against tested bacterial strains, while MBC was calculated as the minimum bactericidal concentration that prevented organism growth after sub-cultured on Muller Hinton agar plate.

### Cancer cell studies

#### Cell culture

Three cancer cell lines, MCF7 (human breast adenocarcinoma), HT29 (human colorectal adenocarcinoma) and HepG2 (human liver adenocarcinoma) were used in this study. All were obtained from the ATCC, USA, and were cultured under standard conditions. The three cancer cells were sub-cultured in RPMI-1640 media (10% FBS); all at 37 °C, 5% CO_2_, and 100% relative humidity, for a maximum of 5–10 passages.

#### Cytotoxicity and selectivity

The cytotoxicity of *B. polystachya* extracts and standard drugs doxorubicin and camptothecin was evaluated using the MTT assay as as previously reported [14, 15]. Cells were treated with varying concentrations of the extracts (0-100 mg/mL)and IC_50_ values were determined. Cell lines were cultured separately and incubated for three days with the extract or doxorubicin at varying concentrations. MTT was added to each well and incubated for three hours at 37 °C. The optical density of the purple formazan was measured at 550 nm absorbance, and the extract concentration causing 50% inhibition (IC_50_) was determined using GraphPad Prism.

## Results and discussion

### GC-MS analysis

**Fig. 1:**
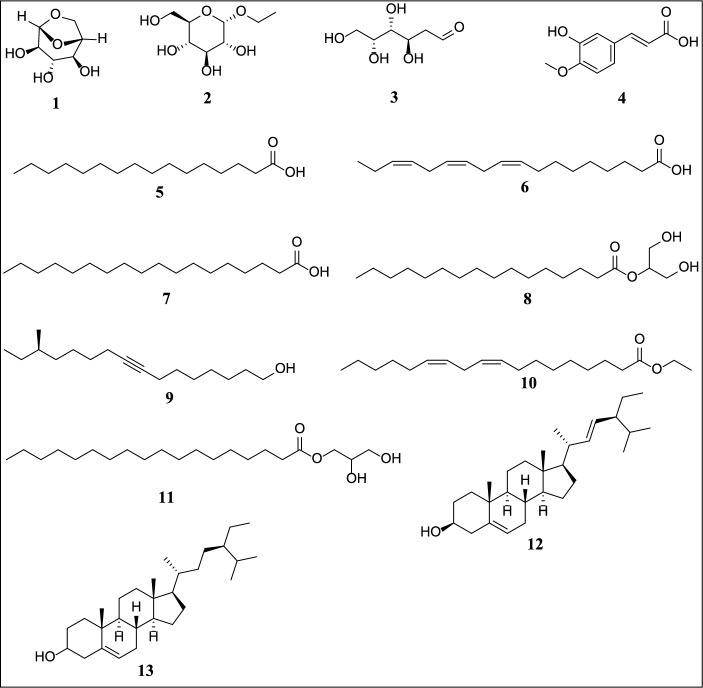
Chemical structures of constituents identified through GC-MS of *Buddleja polystachya* (stem) extracts

The GC-MS analysis revealed a diverse range of 210 compounds in the stem extract and 346 compounds in the leaf extract of *B. polystachya*. The chemicals come from several chemical classes, showing the intricate composition of the plant extracts analyzed. The *B. polystachya* stem extract included high levels of carbohydrates, phenolic compounds, fatty acids, and steroidal components as shown in Table 1; Figs. 1 and 3.

**Table 1:** Phytoconstituents identified by GC‒MS analysis of *Buddleja polystachya* (stem and leaves) extracts

No.	Compound	Molecular formula	Molecular weight	Peak area (%)	Retention time
A. Polystachya stem extracts					
1	1,6-anhydro-beta-d-glucopyranose (levoglucosan)	C_6_H_10_O_5_	162.14	2.42	9.146
2	Ethyl .alpha.-d-glucopyranoside	C_8_H_16_O_6_	208.21	15.98	9.41
3	2-Deoxy-D-galactose	C_6_H_12_O5	164.16	2.44	9.645
4	3-Hydroxy-4-methoxycinnamic acid	C_10_H_10_O4	194.19	2.34	11.214
5	n-Hexadecanoic acid	C_16_H_32_O_2_	256.42	3.87	11.36
6	(Z, Z,Z)-9,12,15-Octadecatrienoic acid	C_18_H_30_O_2_	278.44	2.39	12.527
7	Octadecanoic acid	C_18_H_36_O_2_	284.48	1.81	12.615
8	Hexadecanoic acid, 2-hydroxy-1-(hydroxymethyl)ethyl ester	C_19_H_38_O_4_	330.50	3.24	14.659
9	(R)-(-)-14-Methyl-8-hexadecyn-1-ol	C_17_H_32_O	252.25	2.06	15.729
10	Ethyl Linoleolate	C_20_H_36_O_2_	308.51	1.51	15.8
11	Octadecanoic acid, 2,3-dihydroxypropyl ester	C_21_H_42_O_4_	358.56	3.28	15.868
12	Stigmasterol	C_21_H_42_O_4_	358.56	2.3	22.155
13	Stigmast-5-en-3-ol	C_29_H_50_O	414.71	1.61	23.313

B. polystachya leaves extracts					
1.	D-Allose	C_6_H_12_O_6_	180.06	1.59	8.204
2.	3-Deoxy-d-mannoic lactone	C_6_H_10_O_5_	162.14	1.48	9.003
3.	1,6-anhydro-beta-d-glucopyranose (levoglucosan)	C_6_H_10_O_5_	162.14	1.75	9.134
4.	Ethyl .alpha.-d-glucopyranoside	C_8_H_16_O_6_	208.21	3.17	9.237
5.	n-Hexadecanoic acid	C_16_H_32_O_2_	256.42	1.84	11.342
6.	(Z, Z,Z)-9,12,15-Octadecatrienoic acid	C_18_H_30_O_2_	278.44	1.59	12.516
7.	Octadecanoic acid	C_18_H_36_O_2_	284.48	1.64	12.609
8.	Hexadecanoic acid, 2-hydroxy-1-(hydroxymethyl)ethyl ester	C_19_H_38_O_4_	330.50	2.4	14.654
9.	Phenol, 2-methoxy-4-(1-propenyl)-	C_10_H_12_O_2_	164.20	1.66	15.309
10.	Octadecanoic acid, 2,3-dihydroxypropyl ester	C_21_H_42_O_4_	358.56	5.82	15.865

The chemical classes include carbohydrates (1‒3), phenolic derivatives (4), fatty acids and their derivatives (4‒11), and steroidal components 12 and 13. The prevalence of water-loving carbohydrates 1‒3 are the main elements in this extraction, and when their peak area (%) is greater than 20%, it indicates a potential for water-soluble applications. The stem extract of *B. polystachya* also included fatty acids and their derivatives 4‒11 as the second most abundant components.

Two highly lipophilic steroidal substances, stigmasterol 12 and stigmast-5-en-3-ol 13, were detected in 3.9% of the samples with retention durations exceeding 20 min. The existence of phenolic derivatives and fatty acids suggests a wide range of biological effects, such as antioxidant and anti-inflammatory characteristics. Identifying steroidal components such as stigmasterol and stigmast-5-en-3-ol highlights the possibility for developing drugs based on steroidal scaffolds. The phenolic component 3-hydroxy-4-methoxycinnamic acid was found in this extract. The sugar compound Ethyl.alpha.-d-glucopyranoside (2) was the predominant component in this extract, with a peak area above 15%. Thus, it may be inferred that the stem extract of *B. polystachya* primarily consists of hydrophilic components.

**Fig. 2:**
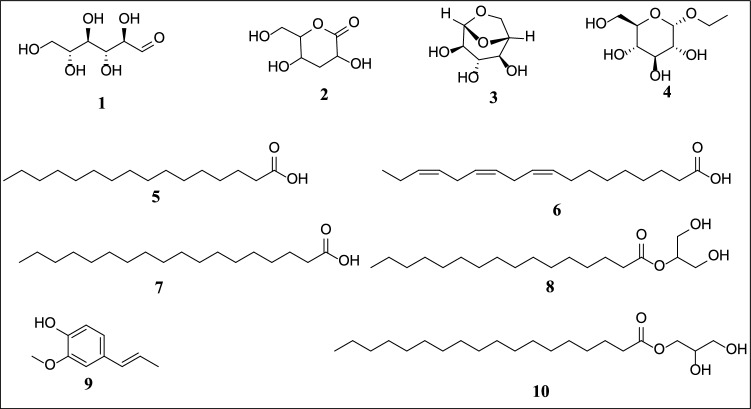
Chemical structures of constituents identified through GC-MS of *Buddleja polystachya* (leaves) extract

**Fig. 3:**
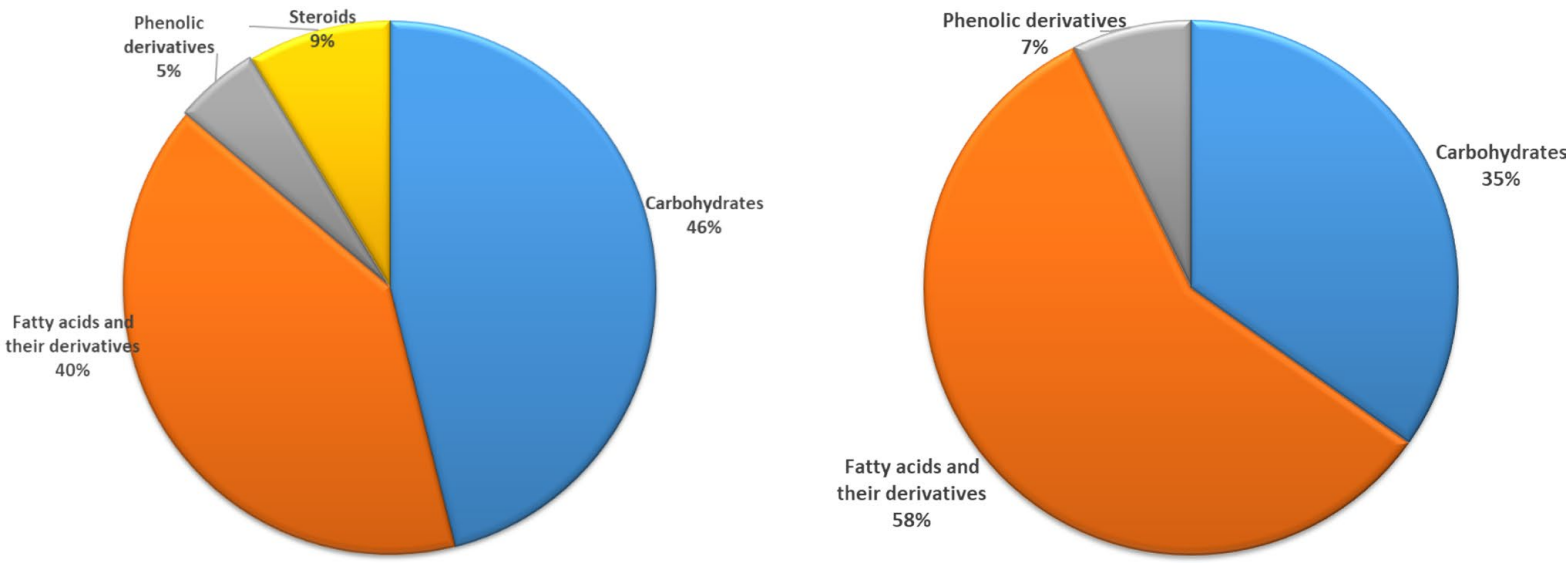
The peak areas% of the major components in *Buddleja polystachya* stem (**A**) and leaves (**B**) extracts

GC-MS profiling of *B. polystachya* leaves extract revealed the presence of 10 main phytochemicals, shown in Table 1; Figs. 2 and 3. The analysis of this extract revealed a combination of hydrophilic carbohydrates and lipophilic fatty acids and their derivatives. The significant amount of octadecanoic acid, 2,3-dihydroxypropyl ester, suggests its possible use in lipid-based formulations or as a bioactive chemical with specific therapeutic capabilities. Carbohydrates (1‒4) make up 7.99% of the total constituents and exhibit peaks with limited retention periods (< 10 min) because of their hydrophilic properties. Fatty acids and their derivatives were the predominant components in the B. *polystachya* (leaves) extract, with a total peak area % above 13%. Due to their elongated hydrocarbon chains, these components exhibited retention durations greater than 11 min. The phenolic component, 9, 2-methoxy-4-(1-propenyl)-phenol, was present in less than 2% abundance. The main phytoconstituent in this extract was identified as octadecanoic acid, 2,3-dihydroxypropyl ester (10), a fatty acid ester with a peak area greater than 5%. This molecule was the most lipophilic among the primary components due to its lengthy hydrophobic chain.

### Antimicrobial studies

Bacterial eye infections attributable to a variety of bacteria, with Gram-positive species being most involved. Symptoms of these infections vary, including blepharitis and conjunctivitis, as well as more serious illnesses such as keratitis, endophthalmitis, and orbital cellulitis. Bacterial isolates’ distribution is regulated by clinical diagnosis and is not limited to certain ocular areas. To effectively manage these infections, a diagnostic approach focused on identifying the cause and a collaborative effort to reduce risks are essential [16, 17].

In this research study, we have also assessed the antibacterial capabilities of *B. polystachya* extracts from the stem and leaves against six microbial strains using the agar diffusion method. The results are detailed in Table 2. The antibacterial effectiveness was measured using inhibition zones (in millimetres ± standard deviation, *n* = 2). Results showed that *B. polystachya* (stem) extracts had a wide range of activity, with inhibition zones ranging from 14 ± 1.0 mm to 18 ± 1.0 mm against *Pseudomonas aeruginosa, Staphylococcus aureus*, and *Streptococcus pneumoniae*. However, there was no activity observed against *Haemophilus influenzae* and *Neisseria gonorrhoeae*. On the other hand, extracts from *B. polystachya* (stem and leaves) showed a similar range of action but were not effective against *S. aureus* and *Candida albicans*, suggesting a limited antibacterial spectrum.

**Table 2:** Antimicrobial effect of *Buddleja polystachya* (stem and leaves) extracts against six microbial strains using agar diffusion assay (diameter, zone of inhibition, mm ± SD, *n* = 2)

B. polystachya extract	Pseudomonas aeruginosa	Staphylococcus aureus	Streptococcus pneumoniae	Haemophilus influenza	Neisseria gonorrhoeae	Candida albicans
Stem extract	14 ± 1.0	17 ± 1.0	18 ± 1.0	11 ± 1.0	R	R
Leaves extract	14 ± 0.0	22 ± 1.0	14 ± 1.0	12 ± 0.0	11 ± 1.0	R
Amikacin	21 ± 0.0	–	–	–	–	–
Vancomycin	–	16 ± 0.0	15 ± 0.0	–	–	–
DMSO cont.	R	R	R	R	R	R

R: resistant, -: not tested,

Amikacin was employed as a positive control for comparison. It exhibited expected inhibitory efficacy against *P. aeruginosa* with a zone of 21 ± 0.0 mm, but its effect on the other strains was not evaluated. Vancomycin showed inhibition zones of 160.0 mm and 150.0 mm against *S. aureus* and *S. pneumoniae*, respectively, in line with its known effectiveness against Gram-positive bacteria. The negative control, DMSO, showed no action against any tested organisms, confirming that the antimicrobial activities reported were due to the active components of the plant extracts and not the solvent.

The antimicrobial assays conducted in this study highlighted t the potential of the extracts under study in combating ocular infections. The variation in the zone of inhibition among different extracts and microbial strains emphasises the specificity and broad-spectrum nature of the antimicrobial compounds present in the extracts.

The determination of MIC and MBC values for the stem and leaves extracts quantifies their efficiency and provides a basis for dose optimization in potential therapeutic applications. Therefore, the antimicrobial properties of *B. polystachya* were further investigated by determination of MICs and MBCs against *Staphylococcus aureus* and *Pseudomonas aeruginosa* (Table 3). The MIC assessments showed similar values for both extracts. The stem extracts showed MICs of 6.2 ± 0.03 mg/mL for *S. aureus* and 6.1 ± 0.05 mg/mL for *P. aeruginosa*. The leaf extracts had MICs of 6.3 ± 0.02 mg/mL and 6.3 ± 0.01 mg/mL against the same bacteria, respectively. The results suggest that the extracts have a strong ability to prevent the growth of bacteria.

**Table 3:** Determination of the MIC and MBC of *Buddleja polystachya* (stem and leaves) extracts (mean ± SD, mg/mL, *n* = 2) against two microbial strains

B. Polystachya Extract	Staphylococcus aureus		Pseudomonas aeruginosa	
	MIC	MBC	MIC	MBC
Stem extract	6.2 ± 0.03	12.1 ± 0.09	6.1 ± 0.05	12.9 ± 0.02
Leaves extract	6.3 ± 0.02	12.5 ± 0.02	6.3 ± 0.01	12.5 ± 0.06

*B. polystachya* stem extracts had MBCs of 12.1 ± 0.09 mg/mL for *S. aureus* and 12.9 ± 0.02 mg/mL for *P. aeruginosa*. *B. polystachya* leaf extracts exhibited MBCs of 12.5 ± 0.02 mg/mL and 12.5 ± 0.06 mg/mL against the same bacteria, respectively. The close proximity of MIC to MBC values for both extracts indicate strong bactericidal effectiveness, capable of not only inhibiting but also completely eliminating bacterial cells at concentrations somewhat higher than the MICs. The low standard deviations highlight the accuracy of these data.

The preliminary findings indicate that the plant extracts, particularly *B. polystachya* (stem) extracts, have significant antibacterial properties that justify further investigation. It is essential to isolate and structurally elucidate the phytochemical components of these extracts to comprehend the factors behind their antibacterial effects. This study confirms the strong antibacterial properties of the plant extracts, suggesting the existence of bioactive chemicals that could be used as templates for developing novel antimicrobial medicines.

### Cytotoxicity and selectivity studies

This research evaluated the cytotoxic effects of the extracts of stems and leaves of *B. polystachya* plant compared to doxorubicin and camptothecin on various cancer cell lines including breast carcinoma (MCF7), colorectal adenocarcinoma (HT29), and hepatocellular carcinoma (HepG2), and normal fibroblast (MRC5). Cytotoxic activities of *B. polystachya* extracts are summarized in Table 4.

**Table 4:** Cytotoxic activity of *Buddleja polystachya* extracts (stem and leaves), doxorubicin and camptothecin against three cell lines, and normal fibroblast (MTT 72 h, IC_50_ ‘’µg/ml’’ ±SD, *n* = 3)

B. polystachya extract	MCF7	HT29	HepG2	IC_50_ (Average*)	MRC5
Stem extract	8.18 ± 0.72	18.20 ± 0.93	25.55 ± 1.42	17.31	4.60 ± 0.05
Leaves extract	34.37 ± 3.43	40.97 ± 0.39	18.38 ± 0.41	31.24	13.14 ± 0.93
Doxo	0.07 ± 0.01	1.98 ± 0.10	2.15 ± 0.15	1.40	5.86 ± 0.35
Campto	0.08 ± 0.01	2.50 ± 0.26	0.76 ± 0.07	1.11	1.18 ± 0.10

^*^Average cytotoxicity (IC_50_) of each extract against the three cancer cells

*B. polystachya* stem extracts showed high to moderate cytotoxicity with IC_50_ values of 8.18 ± 0.72 µg/ml, 18.20 ± 0.93 µg/ml, and 25.55 ± 1.42 µg/ml against MCF7, HT29, and HepG2 cell lines, respectively. *B. polystachya* leaves extract showed moderate to low cytotoxicity in various cancer cell lines, with IC_50_ values of 34.37 ± 3.43 µg/ml for MCF7, 40.97 ± 0.39 µg/ml for HT29, and 18.38 ± 0.41 µg/ml for HepG2. The average IC_50_ values for *B. polystachya* extracts from the stem and leaves against the cancer cell lines were determined to be 17.31 µg/ml and 31.24 µg/ml, respectively. As expected, the used reference compound (doxorubicin) showed stronger cytotoxicity with IC_50_ values of 0.07 ± 0.01 µg/ml, 1.98 ± 0.10 µg/ml, and 2.15 ± 0.15 µg/ml against MCF7, HT29, and HepG2 cell lines, respectively. Camptothecin also exhibited notable cytotoxicity with IC_50_ values of 0.08 ± 0.01 µg/ml, 2.50 ± 0.26 µg/ml, and 0.76 ± 0.07 µg/ml against the cancer cell lines.

### Conclusion and future directions

Extracts of the stems and leaves of *Buddleja polystachya* have significant antimicrobial and cytotoxic potential, according to the study’s results. The phytochemical composition of the sample was determined through GC-MS analysis to be diverse, comprising steroidal components, phenolic derivatives, carbohydrates, and fatty acids. These constituents are responsible for the observed biological activities. The comprehensive results of this investigation regarding the antimicrobial and cytotoxic properties of *B. polystachya* extracts are presented. It emphasizes the notable antibacterial characteristics of the plant, particularly in stem extracts, which are effective against common bacteria that cause ophthalmic infections. Further, the cytotoxic analysis demonstrates the stem extract’s specific effectiveness against various cancer cell lines, thereby emphasizing its potential as a therapeutic agent for cancer. Further research is required to isolate and purify the bioactive constituents of *B. polystachya*, and to test the pure compounds against normal and human microbial flora, other normal cells, and resistant cells and microorganisms, in addition to investigating their mechanism of action including combination studies. Regarding clinical investigations, successful hits would be employed in multiple eye models, including cornea and eye lens. All this could provide a potentially fruitful avenue for the development of innovative antimicrobial and anticancer therapies, specifically for the benefit of ocular health and cancer therapy.

## Data Availability

The entire dataset supporting this work is fully integrated into the publication, guaranteeing transparency and convenient access for additional research and analysis.
